# Supplementary material for: Optogenetic therapy: high spatiotemporal resolution and pattern discrimination compatible with vision restoration in non-human primates
Source: Commun Biol. 2021 Jan 27;4:125. doi: 10.1038/s42003-020-01594-w (PMC7840970; doi:10.1038/s42003-020-01594-w)
Supplement: Supplementary file 2 — Supplementary Information [file 42003_2020_1594_MOESM2_ESM.pdf]

Optogenetic therapy: High spatiotemporal resolution and pattern discrimination compatible with vision restoration in non-human primates

Authors: Gregory Gauvain[1*], Himanshu Akolkar[1,2], Antoine Chaffiol[1], Fabrice Arcizet[1], Mina A. Khoei[1], Mélissa Desrosiers[1], Céline Jaillard[1], Romain Caplette[1], Olivier Marre[1], Stephane Bertin[3], Claire-Maelle Fovet[4], Joanna Demilly[4], Valérie Forster[1], Elena Brazhnikova[1], Philippe Hantraye[4], Pierre Pouget[5], Anne Douar[6], Didier Pruneau[6], Joël Chavas[6], José-Alain Sahel[1,2,3], Deniz Dalkara[1], Jens Duebel[1], Ryad Benosman[1,2], Serge Picaud[1*]

Supplementary Materials

List of Supplementary Materials

Supplementary Materials and Methods

Supplementary Figures

Fig. S1. Clinical evaluation of inflammation after AAV2.7m8 - ChR-tdT treatment

Fig. S2. Total RGC counts in NHP fovea.

Fig. S3.

Fig. S4. Multi-unit activity detection versus the position of the cell identified with a spike-sorting algorithm.

Fig. S5. Almost all the transfected RGCs are midget cells

Fig.S6. Electrode-wise stimulation on a sample retina

Fig.S7. Plane fitting for optical flow calculation.

Table S1. Information about the animals included in the study.

Table S2. Results of Tukey's multiple comparison test for the dose-response effect.

Movie S1. RGC responses to spot stimulation.

Movie S2. RGC responses to moving bars.

Movie S3. RGC responses to different moving shapes.

Supplementary Materials and Methods

AAV production

ChrimsonR and ChrimsonR-tdTomato were inserted into an AAV backbone plasmid. The constructs all included WPRE and bovine growth hormone polyA. Recombinant AAVs were produced by the plasmid cotransfection method[1]. The resulting lysates were purified by iodixanol gradient ultracentrifugation, as previously described. Briefly, the 40% iodixanol fraction was concentrated and subjected to buffer exchange with Amicon Ultra-15 centrifugal

filter units. The DNase-resistant vector genome titers of vector stocks were then determined by real-time PCR relative to a standard[2].

Gene delivery, retina isolation and preservation of the primate retina

Primates were anesthetized with a 10 mg/1 kg mixture of ketamine/xylazine. We injected 100 µL of viral vector solution into the vitreous. An ophthalmic steroid and antibiotic ointment was then applied to the cornea. A lethal dose of pentobarbital was administered two months (± 5 days) or 6 months (± 9 days) after AAV injection. Eyeballs were removed and placed in sealed bags for transport with CO_2-independent medium (Thermo Fisher Scientific), after puncture of the eye with a sterile 20-gauge needle. Retinas were then isolated and conserved as retinal explants in an incubator for 12 to 36 hours before recording. Hemifoveal retinal fragments were transferred onto polycarbonate Transwell inserts (Corning), which were placed on Neurobasal + B27 medium for conservation in the cell culture incubator. In these conditions, natural photoreceptor responses were abolished, and did not recover. We applied pharmacological blockers to check that all natural responses were suppressed (see below).

Two-photon live imaging and single-cell electrophysiological recordings

A custom-made two-photon microscope equipped with a 25x water immersion objective (XLPLN25xWMP, NA: 1.05, Olympus) and a pulsed femto-second laser (InSight™ DeepSee™ - Newport Corporation) was used for imaging ChR-tdT-positive retinal ganglion cells. AAV-treated macaque retinas were imaged in oxygenized (95% O_2, 5% CO_2) Ames medium (Sigma-Aldrich). For live two-photon imaging, whole-mount retinas were placed in the recording chamber of the microscope (ganglion cell layer side up), and images and *z*-stacks were acquired

with an excitation laser used at a wavelength of 1050 nm. Images were processed offline with ImageJ software.

We used an Axon Multiclamp 700B amplifier for whole-cell patch-clamp and cell-attached recordings. Patch electrodes were made from borosilicate glass (BF100-50-10, Sutter Instruments) pulled to 6-9 MΩ. Pipettes were filled with 112.5 mM $CsMeSO_4$, 1 mM Mg SO_4, 7.8 × 10^{-3} mM $CaCl_2$, 0.5 mM BAPTA, 10 mM HEPES, 4 mM ATP-Na_2, 0.5 mM GTP-Na_3, 5 mM lidocaine N-ethyl bromide (QX314-Br) (pH 7.2). We clamped the cells at a potential of -60 mV (the reversal potential of Cl^-), to isolate excitatory currents. Recordings were also made in the cell-attached configuration, with pipettes filled with Ames medium. Retinas were dark-adapted by incubation for at least 30 minutes in the dark, in the recording chamber, before recordings. All the recorded responses were ON responses. We checked that these ON responses originated strictly from ChrimsonR, by making recordings in the presence of an ON-pathway blocker, the selective group III metabotropic glutamate receptor antagonist, L-(+)-2-amino-4-phosphonobutyric acid (L-AP4, 50 μM, Tocris Bioscience, Bristol, UK).

MEA

Multitude electrode array (MEA) recordings were obtained from retinal fragments placed on a cellulose membrane that had been incubated overnight with polylysine (0.1%, Sigma). Once on a micromanipulator, the piece of retina was gently pressed against a MEA (MEA256 100/30 iR-ITO; Multi-Channel Systems, Reutlingen, Germany), with the retinal ganglion cells facing the electrodes. We assessed tdTomato fluorescence, when present, before the recordings, with a Nikon Eclipse Ti inverted microscope (Nikon, Dusseldorf, Germany) mounted under the MEA

system. The retina was continuously perfused with Ames medium (Sigma-Aldrich, St Louis, MO), through which a mixture of 95% O_2 and 5% CO_2 was bubbled at 34 °C, at a rate of 1–2 ml/minute, during experiments. The AMPA/kainate glutamate receptor antagonist 6-cyano-7-nitroquinoxaline-2,3-dione (CNQX, 25 μM, Sigma-Aldrich), the NMDA glutamate receptor antagonist [3H]3-(2-carboxypiperazin-4-yl) propyl-1-phosphonic acid (CPP, 10 μM, Sigma-Aldrich) and a selective group III metabotropic glutamate receptor agonist, L-(+)-2-amino-4-phosphonobutyric acid (L-AP4, 50 μM, Tocris Bioscience, Bristol, UK) were freshly diluted and applied in the bath, via the perfusion system, for 10 minutes before recordings. Action potentials were detected on the filtered electrode signal (2nd order high-pass Butterworth, cut-off frequency 200 Hz) with a threshold of 4 x the SD of the signal. Spike density function, calculated as previously described[3,4], was averaged over repeat simulations and used to measure maximal firing rate in a window corresponding to stimulus duration plus 50 ms. When comparing responses for different vector constructs at different light intensities, we computed, for each electrode, the additional firing rate as the maximal firing rate minus the spontaneous firing rate for this electrode, which is calculated as the mean firing rate in the 2 seconds preceding stimulation.

Photostimulation

For single-cell electrophysiological recordings, a Polychrome V monochromator (Olympus, Hamburg, Germany) set to 600 nm (± 10 nm) was used for photostimulation. Output light intensities were calibrated to range from $5.8x10^{14}$ to $3.15x10^{17}$ photons.$cm^2.s^{-1}$, with a spectrophotometer (USB2000+, Ocean Optics, Dunedin, FL). A customized videoprojector

(Acer DLP P1223, Taiwan) was used in combination with a chromatic filter (590BP20-nm) to generate randomized oscillating stimuli (Fig. 4J).

For MEA recordings, full-field light stimuli were applied with a Polychrome V monochromator (Olympus, Hamburg, Germany) set to 600 nm (± 10 nm), driven by an STG2008 stimulus generator (MCS). Output light intensities were calibrated to range from 1.37 x 10^{14} to 6.78 x 10^{16} photons.cm^2.s^{-1}. For intensity curves, we used two-second flashes for five intensities (1.37 x 10^{14} , 6.56 x 10^{14}, 2.34 x 10^{15}, 8.82 x 10^{15}, 6.78 x 10^{16} photons.cm^2.s^{-1}), each repeated 10 times, with a five-second interstimulus interval. The action spectrum was obtained with a randomized sequence of wavelengths (400 to 650 nm, in 10 nm steps) at constant light power, each repeated 10 times, with a five-second interstimulus interval. High temporal precision and pattern stimulations (moving bar or circular spot) were performed with a digital micromirror display (DMD, Vialux, resolution 1024x768) coupled to a LED fluorescence microscope light source (Xcite, Lumen Dynamics) with a chromatic filter (600BP20-nm), and calibration to deliver a maximum of 2 x 10^{17} photons.cm^2.s^{-1} to the retina. In stimulation duration assays, we used 17 different durations (10, 20, 30, 40, 50, 100, 200, 300, 400, 500, 1000 and 2000 ms), each repeated 10 times, with a five-second interstimulus interval.

Confocal imaging and quantification

After MEA experiments, the tissue was recovered and fixed by incubation for 30 minutes at room temperature (4% PFA), rinsed with PBS and stored at 4°C in sodium azide. Three hemifoveae were used per dose condition, fixed without prior MEA recordings to prevent retinal damage that might impede cell counting. These retinas were fixed on the same day as the MEA

experiments were performed on the same eye and the other hemifovea. Hemifoveas were then mounted on slides in DAPI-containing Vectashield (H-1000, Vector Laboratories) with a coverslip (18 x 18 mm, Biosigma), and a 100 µm spacer (Secure-seal space S24735, Thermo Fisher Scientific), and the slides were sealed with nail polish. The retinas were imaged on an inverted confocal microscope (Fluoview 1200, Olympus), with a 20X objective (UPLSAPO 20XO, NA: 0.85, Olympus), a voxel size of 0.265 to 0.388 µm/pixel for x and y and 1.64 µm/pixel for z. For each hemifovea, multiple stacks were registered and reconstituted in an automatic stitch (10% overlap). Using Td-tomato fluorescence, we performed blind manual 3D counts of the transfected cells in the ImageJ (http://imagej.nih.gov/ij) cell counter plugin. The results were processed with custom-built matlab analysis software, for calculations of *local density*, the *density relative to eccentricity* and *averaged total transfected cells*. *Local density* was calculated by binning on cell coordinates, with individual bin sizes as close as possible to 50 µm^2 and a whole number of bins for every stitch. The *density relative to eccentricity* was calculated from the Euclidean distances between each counted cell and the center of the fovea. We estimated the position of the center of the fovea from the center of a disk drawn to represent the shape of the fovea. Our retina explants were cut to obtain hemifoveae on two explants, but the cut was not at 180°. For each explant, we estimated the angle covered by the retina explants, using the calculated center of the fovea, and used it to estimate the total density of ChR-tdT-expressing RGCs relative to eccentricity.

The estimated *averaged total transfected cells* number was calculated as the total number of counted ChR-tdT-positive cells in the explant, divided by the angle of the fovea in the explant and multiplied by 360 degrees.

Descriptive statistics for population responses on multielectrode arrays

In analyses of response patterns for stimuli of increasing duration (1 ms to 2000 m, Fig. 3) we calculated the *time to first spike* and *Fano factor*. Time to first spike was determined as the time between the onset of stimulation and the next spike on the electrode. For each electrode, this value was averaged over all repeats for a specific duration. We then constructed a box-plot for each duration, for all electrodes in all experiments. The Fano Factor, *F*, was calculated for each electrode as:

$$F = \frac{< \delta N^2 >}{< N >}$$

Where $< \delta N^2 >$ is the mean spike variance, and is divided by $< N >$, the mean spike count.

Letter discrimination based on the population response

Three different shapes (X, circle and square) were presented over the retina, moving in a center-out configuration, in eight directions. The shapes were created with Snellen chart ratios, such that the ratio of width of the individual edges of the shape to the total size was 1:5. Widths of 11 µm to 66 µm were used, creating shapes of 55 µm to 330 µm. Each condition was presented over 50 trials. The response of the population of cells to each representation could be represented as a set of K spikes $S = \{S_1, S_2, S_k \ldots S_K\}$. Each spike S_k is a two-dimensional word $\{i, t_k\}$, representing a spike for cell i at time t_k.

The spike train S can be considered as a population signature representing each presented stimulus. These spike patterns S were used for pattern discrimination resembling an algorithm[70] developed for event-trains from neuromorphic sensors. The intercell spike intervals were used to create temporal features. For each set S, a feature vector $F \in R^N$ (N = Number of cells) was created by averaging the temporal feature vectors f for each spike S_k (Eq 1).

$$f_{k,i} = e^{-\frac{|t_k - t_i|}{\tau}} (1)$$

$$F(i) = \frac{1}{K}\sum_{k=1}^{K} f_{k,i} \ (2)$$

where t_k is the spike time for S_k and t_i is the last spike at every other cell i and τ is the decay constant dictating the effect of the temporal history of the last spike of the cell (set to 5 ms in this case). Thus, each trial T of a shape is represented by the vector F. Finally, a k-means-based linear discriminator, trained over half the trials, was used to discriminate between the three shapes in the remaining half of the trials. The performance of the feature and k-means method was quantified as the information common to the presented and predicted stimulus shapes, $I(L; L^p)$, (Eq 3)[5] where $P(L,L^p)$ represents the joint probabilities for a presented shape L predicted by the decoder as L^p.

$$I(L; L^p) = \sum_{L,L^p} P(L, L^p) log_2 \frac{P(L,L^p)}{P(L)P(L^p)} (3)$$

This measurement is more useful than average discrimination accuracy, as it also incorporates cross-stimulus errors. Indeed, it quantifies both the closeness of the features within the same class cluster and their distance from the features of all other classes. This calculation was performed with the matlab information breakdown toolbox[62].

Moving bar stimulation and direction estimation

The retina was stimulated with moving bars at two speeds (2.2 mm/s, 4.4 mm/s), in four directions (0, 45, 90, 135°; Fig. 8). In each trial, the bar was moved over the whole retina. We recorded 200 trials for each condition. Each trial created a three-dimensional spike pattern in which each spike could be represented as $R^3 = \{x, y, t\}$ where $[x, y]$ are the coordinates representing MEA electrode location and t is the timing of the spike since the start of bar motion (Fig. S7). A simple least squares estimate method was used to fit a plane (Eq. 4). The partial and composite slopes from the coefficients of the plane provided both the speed and direction of the bar if the temporal precision of the spikes generated was sufficiently high (i.e. the cells were able to respond faster than the bar could move from one cell to the next).

$$Ax + By + Ct + D = 0 \quad (4)$$

$$\frac{\delta x}{\delta t} \equiv \frac{-C}{A}, \frac{\delta y}{\delta t} \equiv \frac{-C}{B} \; and \; \frac{\delta x}{\delta y} \equiv \frac{-B}{A} \quad (5)$$

.

Supplementary Figures

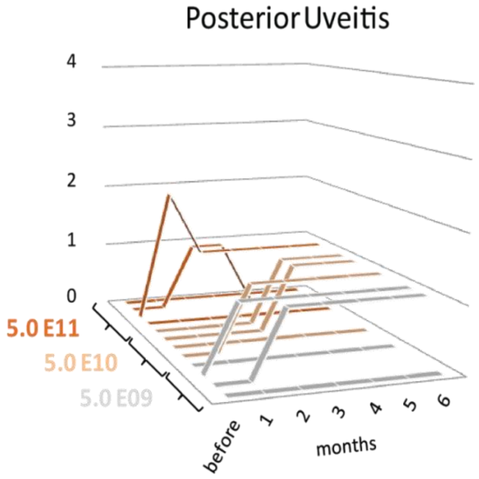

Grade	Clinical findings
0	No vitreous cells
1	Vitreous cells with clear view of posterior fundus
2	Vitreous cells with poor view of details, and view of the optic nerve and retinal vessel
3	Vitreous cells with view of optic nerve
4	Vitreous cells with no view of the posterior segment

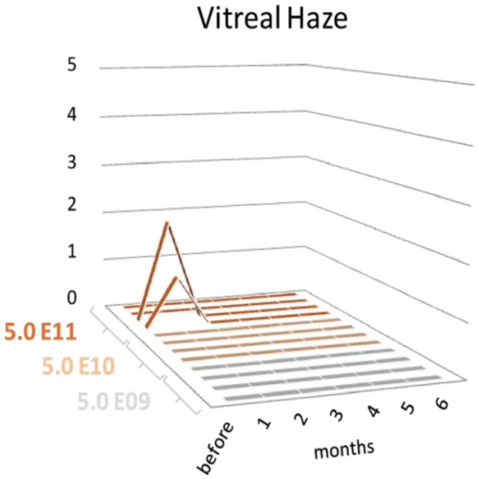

Grade	Clinical findings
0	None
1	Posterior pole clearly visible
2	Posterior pole details slightly hazy
3	Posterior pole details very hazy
4	Posterior pole details barely visible
5	Fundal details not visible

Fig. S1. Clinical evaluation of inflammation after AAV2.7m8-ChR-tdT treatment

(***top***) Examination for posterior uveitis according to the grades indicated on the right. Grade 2 uveitis was observed in only one animal (treated with a high dose of vector), and only for the first month. (***bottom***) Assessment of vitreal haze. Only two animals presented signs of haze. The haziest fundus was that of the animal presenting grade 2 uveitis. The haze dissipated two months

after the injection, in both animals. These two observations indicate a mild reaction to the intravitreous injection (this particular animal presented a small hemorrhage when the sclera was pierced). The expression of an ectopic transgene usually reaches full potency at two months of expression, and there were no signs of delayed inflammation.

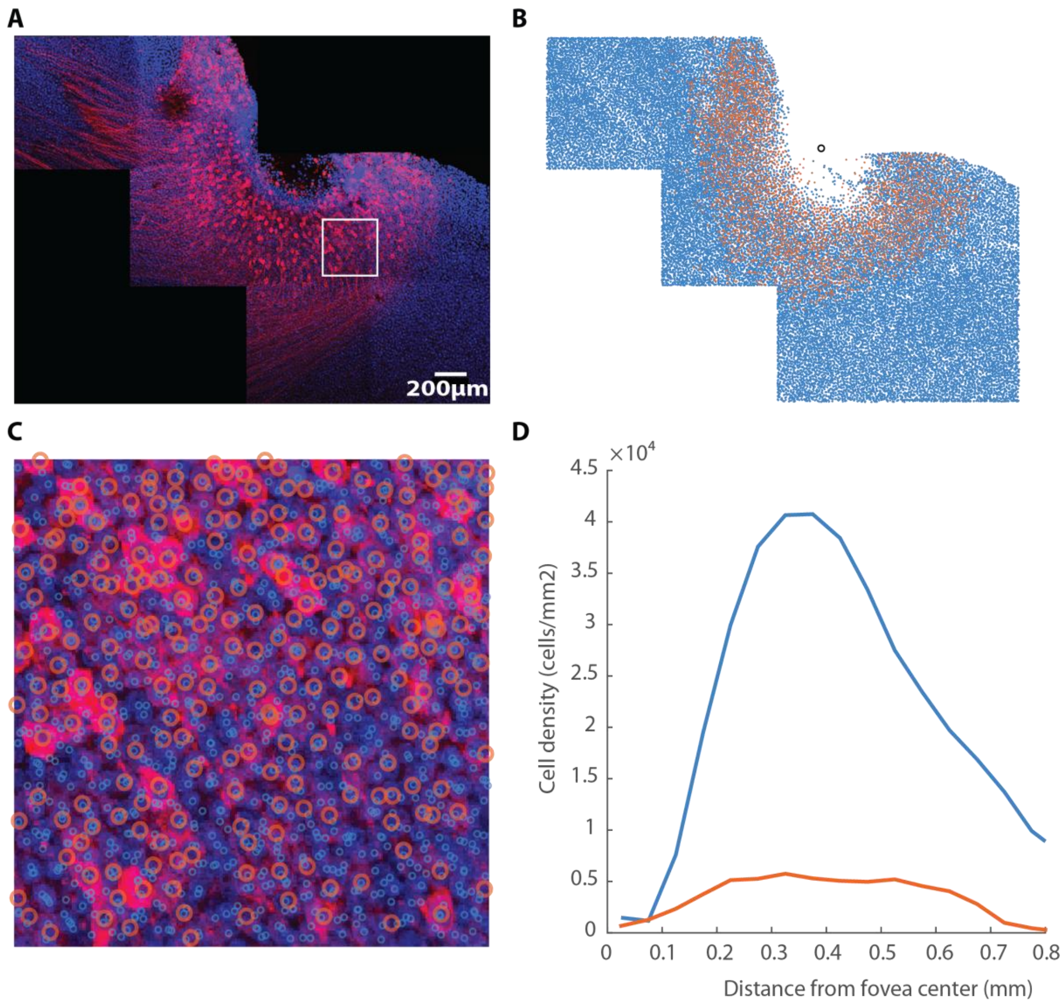

Fig. S2. Total RGC count in the NHP fovea.

(A) Projection of stitched confocal stacks of a DAPI-stained ChR-tdT-expressing fovea. The assembled stitch was edited to remove nuclei below the RGC layer. **(B)** Overlay of manually

counted ChR-tdT-expressing RGCs and automated nucleus detection with Imaris (see supplementary methods) for the complete stitch. The black circle represents the estimated fovea center. **(C)** Close-up of the white square shown in (A), open orange circles depict ChR-tdT-positive RGCs, blue circles are nuclei labeled with DAPI. (D) Total cell density in the RGC layer relative to retina eccentricity, obtained from DAPI nucleus counts (blue line). Note that density peaks at about 40 000 cells/mm² at 0.4 mm eccentricity. The density of ChR-tdT-positive cells relative to retina eccentricity is provided for comparison (red line), This particular set of images was obtained with a retina receiving a medium dose (5×10^{10} vg/eye), and shows a maximal density of ~5000 cells/mm² (~12.5% of total cells).

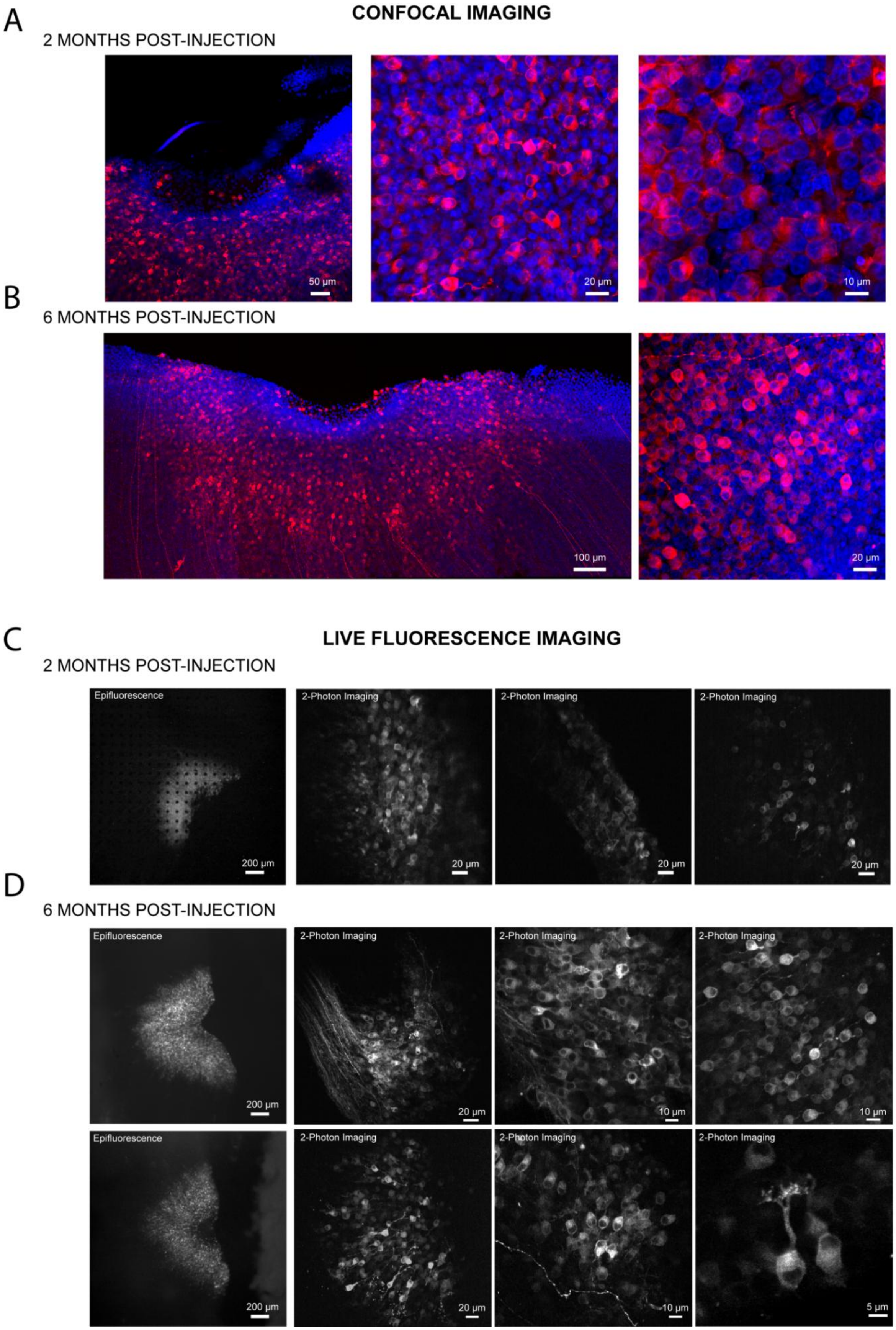

A
CONFOCAL IMAGING
2 MONTHS POST-INJECTION
50 µm
20 µm
10 µm
B
6 MONTHS POST-INJECTION
100 µm
20 µm
C
LIVE FLUORESCENCE IMAGING
2 MONTHS POST-INJECTION
Epifluorescence
2-Photon Imaging
200 µm
20 µm
D
6 MONTHS POST-INJECTION
10 µm
5 µm

Fig. S3. Morphological comparison at 2 and 6 months after AAV2.7m8-ChR-tdT treatment

(A-B) Confocal stack projections comparing ChR-tdT expression in the fovea of animals at 2 months **(A)** or 6 months **(B)** after intravitreal injection. Red: ChR-tdT. Blue: DAPI nuclear labeling. **(C-D)** Live imaging, by epifluorescence (left images) or two-photon imaging (middle and right images), comparing ChR-tdT expression in the foveae of animals at 2 months **(A)** or 6 months **(B)** after intravitreal injections.

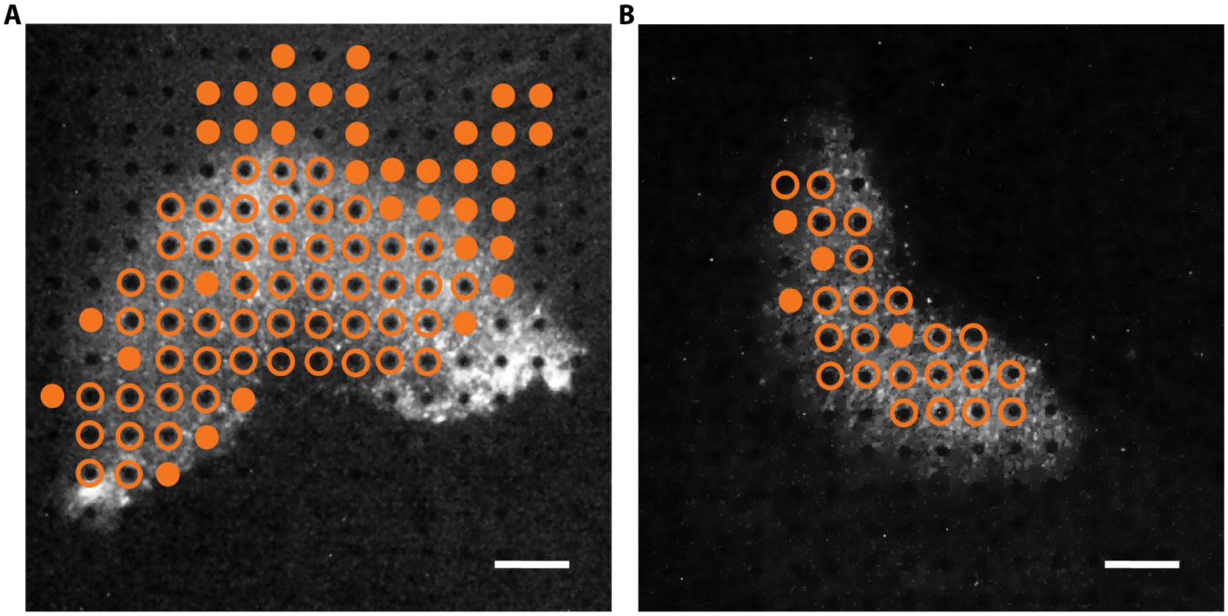

Fig. S4. Multi-unit activity detection versus position of the cell identified with a spike-sorting algorithm

(A-B) Recording examples for retinas treated with high dose, yielding high functional efficacy (A) or moderate functional efficacy (B).Open orange circles represent electrodes at which at least one neuron was identified by spike sorting, whereas filled circles represent electrodes at which multi-unit spiking activity from neurons located at the open circles was detected (threshold of 4x the SD of the signal before stimulation).Scale bar: 200 µm.

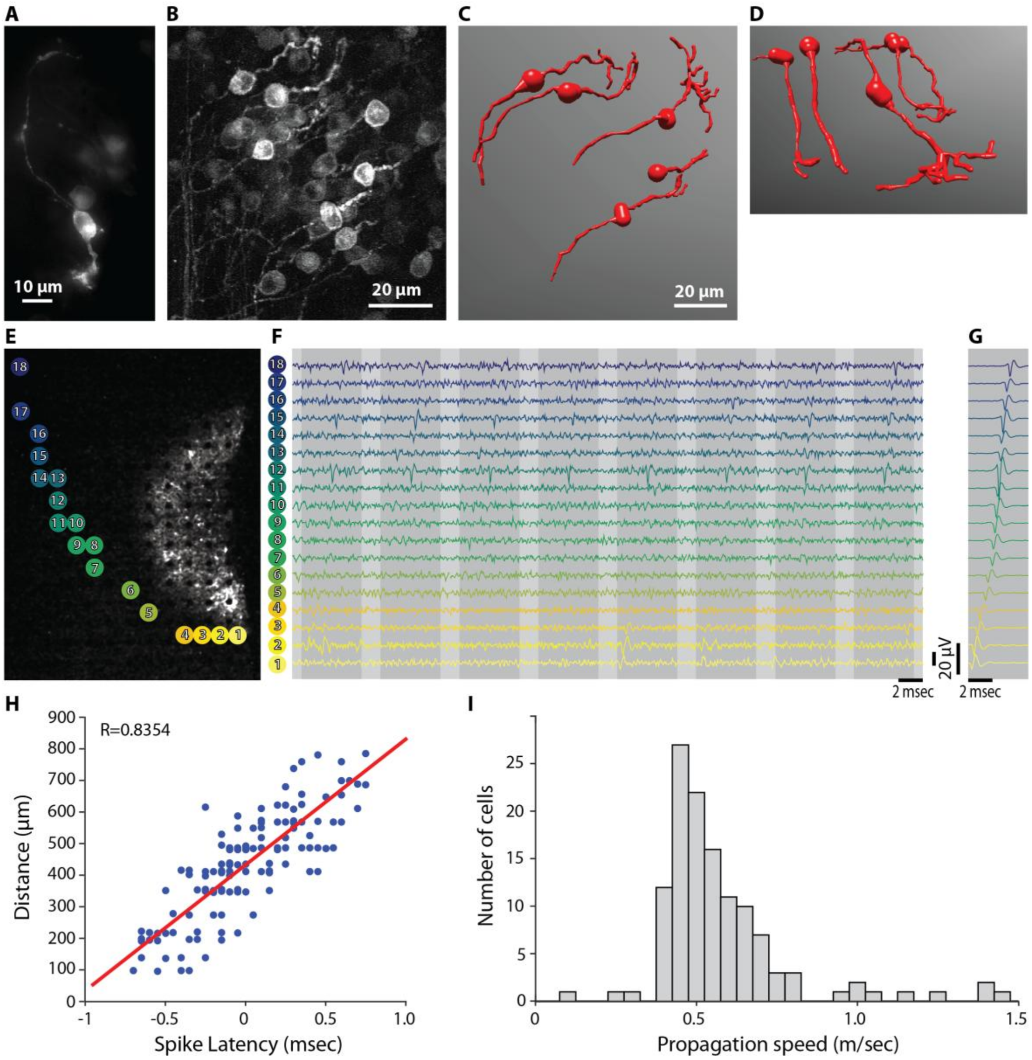

Fig. S5. Almost all the transfected RGCs are midget cells

(**A**) Epifluorescence image of a ChR-tdT-expressing RGC close to the fovea Its morphology is typical of midget ganglions: a small soma (circle) with a single dendrite (arrow) and little or no terminal ramification. The axon can be seen on the opposite side of the cell (arrowhead). (**B**) Projection of a stack of two-photon images displaying a cluster of RGCs with strong ChR-tdT expression. (**C-D**) Bird's-eye view (C) and side view (D) of a 3D reconstruction of the complete RGCs present in the images. Four of the cells displayed very restricted terminal dendritic arborization (5-10 µm), and the fifth cell had a more extensive arborization (~40 µm),

corresponding to midget ganglion cells and parasol ganglion cells, respectively. Note that the soma of the putative parasol cell lies deeper in the ganglion cell layer. (**E-I**) Measurement of spike propagation speed in responsive RGCs expressing ChR-tdT. (**E**) Image of a hemifovea on the MEA overlaid with the position of the electrodes displaying the responses of individual RGCs, numbered 1 to 18, with 1 closest to the soma and 18 furthest away. (**F-G**) Traces showing responses in 8 trials (F, dark gray), or the mean response (G) to circular stimulation via electrode 1. In the mean response, a progressive increase in spike latency is clearly visible. (**H**) Linear regression analysis confirmed the positive association of electrode distance from the site of spike initiation (soma) with recorded latency at that electrode. The slope of the linear regression line was used to estimate the speed of action potential propagation for that ganglion cell. (**I**) Histogram of propagation speed in our population. Most of the cells have a propagation speed of about 0.5 m/s, confirming that they are indeed midget ganglion cells *(32)*. Few cells have a propagation speed greater than 1 m/s and these cells are probably parasol cells.

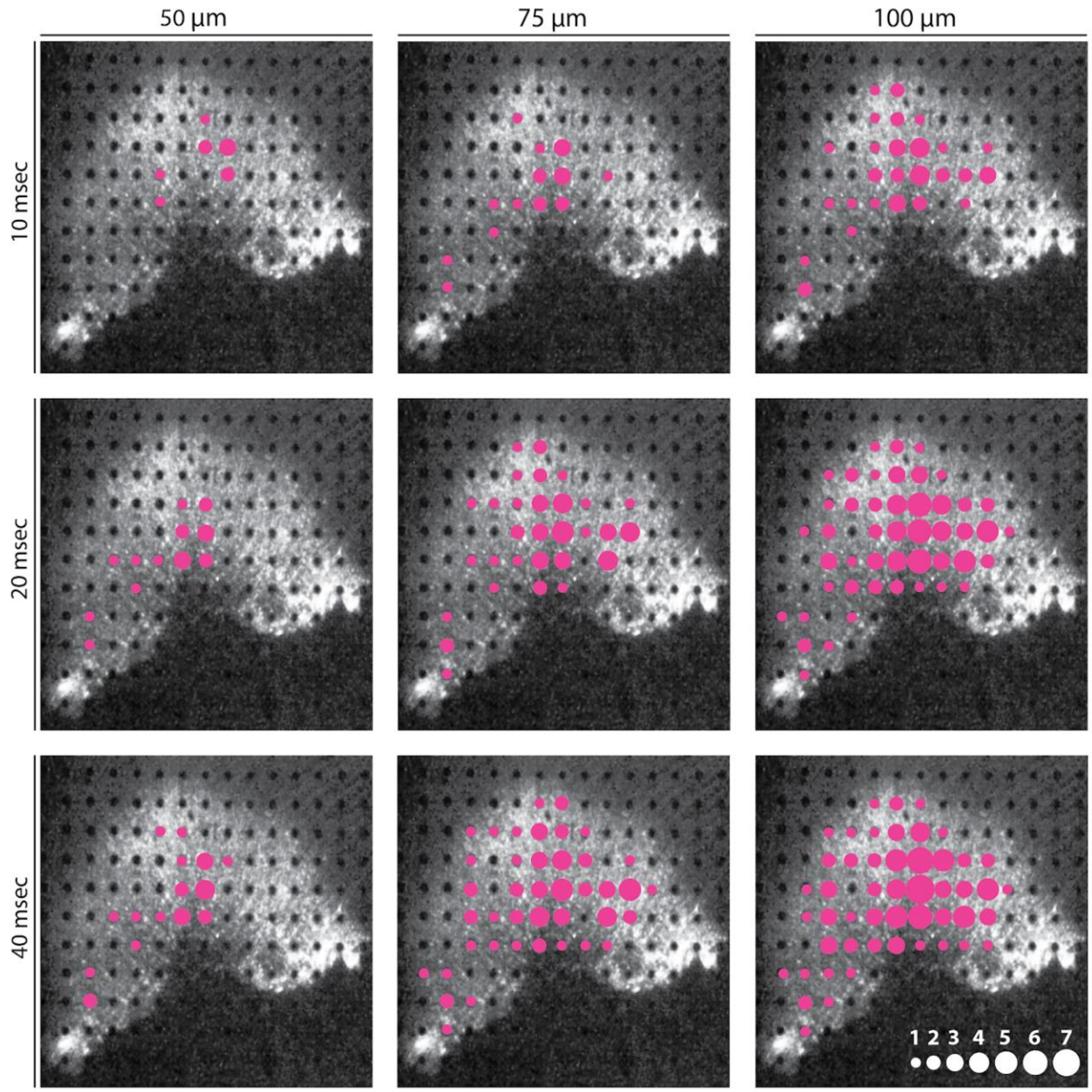

Fig. S6. Electrode-wise stimulation on a sample retina

Circular spots of light of various sizes (50-75-100 µm) were presented at the electrode sites for various durations (10-20-40 ms). Each line of images corresponds to a different duration of stimulation, and each column corresponds to a different stimulus size. The number of cells

recruited per stimulated electrode is represented as a magenta disk of variable size (see scale in the bottom right corner).

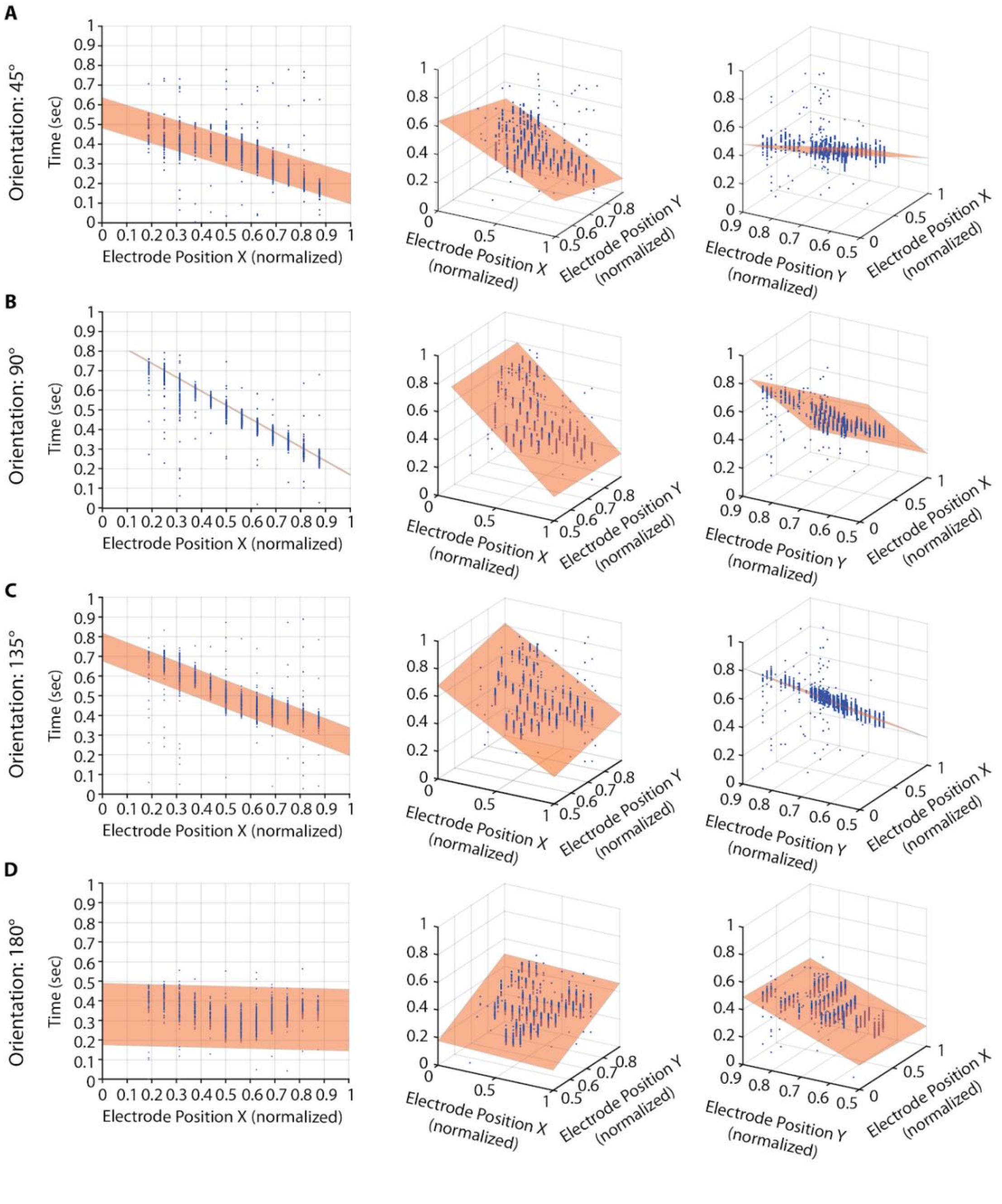

Fig. S7. Plane fitting for optical flow calculation

Four different directions of movement are shown (**A**: 45°, **B**: 90°, **C**: 135°, **D**: 180°). Cell normalized time for peak firing rate is indicated relative to the position of the main electrode recording the cell concerned (blue dots). The 2D plane fitting the peak activity timing is displayed in orange. Three orientations for the same plot are shown here to improve visualization of the change in the fitted plane (0° rotation on the left, -30° rotation in the middle, and 60° rotation on the right).

NHP number	Sex	Age (years)	Right eye		Left eye		Expression duration (days)
			Viral genome/ eye	Construct	Viral genome/ eye	Virus	
CA662	M	3	5.00E+11	AAV2-7m8-ChrimsonR	5.00E+11	AAV2-7m8-ChrimsonR-tdTomato	55
CA680	M	4	5.00E+11	AAV2-7m8-ChrimsonR	5.00E+11	AAV2-7m8-ChrimsonR-tdTomato	57
BX914	M	4	5.00E+11	AAV2-7m8-ChrimsonR	5.00E+11	AAV2-7m8-ChrimsonR-tdTomato	62
BX430	M	4	5.00E+11	AAV2-7m8-ChrimsonR	5.00E+11	AAV2-7m8-ChrimsonR-tdTomato	63
CB206	F	7	5.00E+11	AAV2-ChrimsonR	5.00E+11	AAV2-ChrimsonR-tdTomato	55
CA295	F	13	5.00E+11	AAV2-ChrimsonR	5.00E+11	AAV2-ChrimsonR-tdTomato	56
CB737	M	3	5.00E+11	AAV2-ChrimsonR	5.00E+11	AAV2-ChrimsonR-tdTomato	62
P2	M	3	5.00E+11	AAV2-ChrimsonR	5.00E+11	AAV2-ChrimsonR-tdTomato	64
BA586I	M	4	5.00E+10	AAV2-7m8-ChrimsonR-tdTomato	5.00E+11	AAV2-7m8-ChrimsonR-tdTomato	182
BB161I	M	5	5.00E+09	AAV2-7m8-ChrimsonR-tdTomato	5.00E+10	AAV2-7m8-ChrimsonR-tdTomato	184
BB478E	M	4	5.00E+09	AAV2-7m8-ChrimsonR-tdTomato	5.00E+10	AAV2-7m8-ChrimsonR-tdTomato	182
BA901H	M	4	5.00E+11	AAV2-7m8-ChrimsonR-tdTomato	5.00E+10	AAV2-7m8-ChrimsonR-tdTomato	184
BB222I	M	4	5.00E+09	AAV2-7m8-ChrimsonR-tdTomato	5.00E+11	AAV2-7m8-ChrimsonR-tdTomato	190
BC110B	M	5	5.00E+11	AAV2-7m8-ChrimsonR-tdTomato	5.00E+09	AAV2-7m8-ChrimsonR-tdTomato	191
1107044	F	5	5.00E+11	AAV2-7m8-ChrimsonR-tdTomato	5.00E+11	AAV2-7m8-ChrimsonR-tdTomato	177
1107086	F	5	5.00E+11	AAV2-7m8-ChrimsonR-tdTomato	5.00E+11	AAV2-7m8-ChrimsonR-tdTomato	175
1106077	M	5	5.00E+11	AAV2-7m8-ChrimsonR-tdTomato	5.00E+11	AAV2-7m8-ChrimsonR-tdTomato	161
BB325G	M	4	5.00E+11	AAV2-7m8-ChrimsonR-tdTomato	5.00E+11	AAV2-7m8-ChrimsonR-tdTomato	163

Table S1. Information about the animals included in the study.

NHP number: identification number for non human primates (*macaca fascicularis*) included in the study. 18 animals in total were included (4 females, 14 males), age ranging from 3 to 13 years old. Details of the eye treatment are given as a dose and corresponding construct used.

Expression duration: 55 to 64 days for the 2months experiments, and 161 to 191 days for the 6 months experiments.

		Intensity 4 (8.82×10^{15} photons.cm^{-2}.s^{-1})				Intensity 5 (6.78×10^{16} photons.cm^{-2}.s^{-1})			
Dose	Tukey's multiple comparisons test	Mean Diff	95.00% CI of diff	Summary	Adjusted P Value	Mean Diff2	95.00% CI of diff3	Summary4	Adjusted P Value5
5×10^{9} vg/eye	(5E09).BB22i_OD vs. (5E10).BA586i_OD	-2.599	-57.12 to 51.93	ns	>0.9999	-36.39	-90.91 to 18.14	ns	0.563
	(5E09).BB22i_OD vs. (5E10).BB478_OG1	-0.1387	-56.53 to 56.26	ns	>0.9999	-3.324	-59.72 to 53.07	ns	>0.9999
	(5E09).BB22i_OD vs. (5E10).BB161i_OG	-8.784	-61.72 to 44.15	ns	>0.9999	-125	-178 to -72.1	****	<0.0001
	(5E09).BB22i_OD vs. (5E11).BA901h_OD	-20.19	-73.67 to 33.28	ns	0.9862	-215.3	-268.7 to -161.8	****	<0.0001
	(5E09).BB22i_OD vs. (5E11).BB222i_OG	-19.05	-72.3 to 34.2	ns	0.9911	-118.3	-171.6 to -65.1	****	<0.0001
	(5E09).BB22i_OD vs. (5E11).1106077_OD	-74.26	-128.6 to -19.88	***	0.0005	-230.9	-285.3 to -176.6	****	<0.0001
	(5E09).BB22i_OD vs. (5E11).1107086_OD	-116.9	-171.3 to -62.43	****	<0.0001	-254.4	-308.8 to -199.9	****	<0.0001
	(5E09).BB22i_OD vs. (5E11).1107086_OG	-10.1	-75.06 to 54.86	ns	>0.9999	-42.49	-107.4 to 22.48	ns	0.595
	(5E09).BB22i_OD vs. (5E11).1106077_OG	-4.953	-64.07 to 54.16	ns	>0.9999	-63.51	-122.6 to -4.388	*	0.0228
	(5E09).BB22i_OD vs. (5E11).BB325g_OD	-73.66	-134 to -13.36	**	0.0038	-202.7	-263 to -142.4	****	<0.0001
	(5E09).BB22i_OD vs. (5E11).BB325g_OG	-2.548	-71.26 to 66.16	ns	>0.9999	-42.59	-111.3 to 26.12	ns	0.675
Dose	Tukey's multiple comparisons test	Mean Diff	95.00% CI of diff	Summary	Adjusted P Value	Mean Diff2	95.00% CI of diff3	Summary4	Adjusted P Value5
5×10^{10} vg/eye	(5E10).BA586i_OD vs. (5E10).BB478_OG1	2.46	-29.94 to 34.86	ns	>0.9999	33.06	0.6603 to 65.46	*	0.0406
	(5E10).BA586i_OD vs. (5E10).BB161i_OG	-6.186	-32.11 to 19.74	ns	0.9998	-88.65	-114.6 to -62.73	****	<0.0001
	(5E10).BA586i_OD vs. (5E11).BA901h_OD	-17.6	-44.59 to 9.402	ns	0.6003	-178.9	-205.9 to -151.9	****	<0.0001
	(5E10).BA586i_OD vs. (5E11).BB222i_OG	-16.45	-43 to 10.09	ns	0.6754	-81.96	-108.5 to -55.41	****	<0.0001
	(5E10).BA586i_OD vs. (5E11).1106077_OD	-71.66	-100.4 to -42.91	****	<0.0001	-194.5	-223.3 to -165.8	****	<0.0001
	(5E10).BA586i_OD vs. (5E11).1107086_OD	-114.3	-143.2 to -85.4	****	<0.0001	-218	-246.9 to -189.1	****	<0.0001
	(5E10).BA586i_OD vs. (5E11).1107086_OG	-7.5	-53.21 to 38.21	ns	>0.9999	-6.1	-51.81 to 39.61	ns	>0.9999
	(5E10).BA586i_OD vs. (5E11).1106077_OG	-2.354	-39.29 to 34.58	ns	>0.9999	-27.12	-64.05 to 9.816	ns	0.4056
	(5E10).BA586i_OD vs. (5E11).BB325g_OD	-71.06	-109.9 to -32.26	****	<0.0001	-166.3	-205.1 to -127.5	****	<0.0001
	(5E10).BA586i_OD vs. (5E11).BB325g_OG	0.05052	-50.84 to 50.94	ns	>0.9999	-6.206	-57.1 to 44.69	ns	>0.9999
	(5E10).BB478_OG1 vs. (5E10).BB161i_OG	-8.646	-38.3 to 21.01	ns	0.9985	-121.7	-151.4 to -92.06	****	<0.0001
	(5E10).BB478_OG1 vs. (5E11).BA901h_OD	-20.06	-50.66 to 10.54	ns	0.5916	-211.9	-242.5 to -181.3	****	<0.0001
	(5E10).BB478_OG1 vs. (5E11).BB222i_OG	-18.91	-49.12 to 11.29	ns	0.6606	-115	-145.2 to -84.82	****	<0.0001
	(5E10).BB478_OG1 vs. (5E11).1106077_OD	-74.12	-106.3 to -41.96	****	<0.0001	-227.6	-259.8 to -195.5	****	<0.0001
	(5E10).BB478_OG1 vs. (5E11).1107086_OD	-116.7	-149 to -84.47	****	<0.0001	-251	-283.3 to -218.8	****	<0.0001
	(5E10).BB478_OG1 vs. (5E11).1107086_OG	-9.96	-57.89 to 37.97	ns	>0.9999	-39.16	-87.09 to 8.767	ns	0.2408
	(5E10).BB478_OG1 vs. (5E11).1106077_OG	-4.814	-44.46 to 34.83	ns	>0.9999	-60.18	-99.83 to -20.54	****	<0.0001
	(5E10).BB478_OG1 vs. (5E11).BB325g_OD	-73.52	-114.9 to -32.13	****	<0.0001	-199.4	-240.8 to -158	****	<0.0001
	(5E10).BB478_OG1 vs. (5E11).BB325g_OG	-2.409	-55.3 to 50.48	ns	>0.9999	-39.27	-92.16 to 13.63	ns	0.3875
	(5E10).BB161i_OG vs. (5E11).BA901h_OD	-11.41	-35.04 to 12.22	ns	0.917	-90.22	-113.9 to -66.59	****	<0.0001
	(5E10).BB161i_OG vs. (5E11).BB222i_OG	-10.27	-33.38 to 12.85	ns	0.9529	6.691	-16.42 to 29.81	ns	0.9986
	(5E10).BB161i_OG vs. (5E11).1106077_OD	-65.47	-91.09 to -39.86	****	<0.0001	-105.9	-131.5 to -80.28	****	<0.0001
	(5E10).BB161i_OG vs. (5E11).1107086_OD	-108.1	-133.9 to -82.33	****	<0.0001	-129.3	-155.1 to -103.6	****	<0.0001
	(5E10).BB161i_OG vs. (5E11).1107086_OG	-1.314	-45.12 to 42.49	ns	>0.9999	82.55	38.74 to 126.4	****	<0.0001
	(5E10).BB161i_OG vs. (5E11).1106077_OG	3.831	-30.72 to 38.38	ns	>0.9999	61.53	26.98 to 96.08	****	<0.0001
	(5E10).BB161i_OG vs. (5E11).BB325g_OD	-64.88	-101.4 to -28.34	****	<0.0001	-77.7	-114.2 to -41.15	****	<0.0001
	(5E10).BB161i_OG vs. (5E11).BB325g_OG	6.236	-42.95 to 55.43	ns	>0.9999	82.45	33.25 to 131.6	****	<0.0001
Dose	Tukey's multiple comparisons test	Mean Diff	95.00% CI of diff	Summary	Adjusted P Value	Mean Diff2	95.00% CI of diff3	Summary4	Adjusted P Value5
5×10^{11} vg/eye	(5E11).BA901h_OD vs. (5E11).BB222i_OG	1.143	-23.17 to 25.46	ns	>0.9999	96.91	72.6 to 121.2	****	<0.0001
	(5E11).BA901h_OD vs. (5E11).1106077_OD	-54.06	-80.77 to -27.36	****	<0.0001	-15.67	-42.38 to 11.03	ns	0.7475
	(5E11).BA901h_OD vs. (5E11).1107086_OD	-96.69	-123.5 to -69.84	****	<0.0001	-39.11	-65.95 to -12.26	***	0.0001
	(5E11).BA901h_OD vs. (5E11).1107086_OG	10.1	-34.36 to 54.55	ns	0.9999	172.8	128.3 to 217.2	****	<0.0001
	(5E11).BA901h_OD vs. (5E11).1106077_OG	15.24	-20.12 to 50.61	ns	0.962	151.8	116.4 to 187.1	****	<0.0001
	(5E11).BA901h_OD vs. (5E11).BB325g_OD	-53.47	-90.78 to -16.16	***	0.0002	12.53	-24.78 to 49.84	ns	0.9948
	(5E11).BA901h_OD vs. (5E11).BB325g_OG	17.65	-32.12 to 67.41	ns	0.9918	172.7	122.9 to 222.4	****	<0.0001
	(5E11).BB222i_OG vs. (5E11).1106077_OD	-55.21	-81.46 to -28.96	****	<0.0001	-112.6	-138.8 to -86.34	****	<0.0001
	(5E11).BB222i_OG vs. (5E11).1107086_OD	-97.83	-124.2 to -71.44	****	<0.0001	-136	-162.4 to -109.6	****	<0.0001
	(5E11).BB222i_OG vs. (5E11).1107086_OG	8.952	-35.23 to 53.13	ns	>0.9999	75.86	31.68 to 120	****	<0.0001
	(5E11).BB222i_OG vs. (5E11).1106077_OG	14.1	-20.92 to 49.12	ns	0.9772	54.84	19.82 to 89.86	****	<0.0001
	(5E11).BB222i_OG vs. (5E11).BB325g_OD	-54.61	-91.6 to -17.63	****	<0.0001	-84.39	-121.4 to -47.4	****	<0.0001
	(5E11).BB222i_OG vs. (5E11).BB325g_OG	16.5	-33.02 to 66.03	ns	0.9952	75.75	26.23 to 125.3	****	<0.0001
	(5E11).1106077_OD vs. (5E11).1107086_OD	-42.62	-71.23 to -14.01	****	<0.0001	-23.43	-52.04 to 5.177	ns	0.2375
	(5E11).1106077_OD vs. (5E11).1107086_OG	64.16	18.62 to 109.7	***	0.0003	188.4	142.9 to 234	****	<0.0001
	(5E11).1106077_OD vs. (5E11).1106077_OG	69.31	32.58 to 106	****	<0.0001	167.4	130.7 to 204.1	****	<0.0001
	(5E11).1106077_OD vs. (5E11).BB325g_OD	0.5955	-38 to 39.2	ns	>0.9999	28.2	-10.4 to 66.8	ns	0.4138
	(5E11).1106077_OD vs. (5E11).BB325g_OG	71.71	20.97 to 122.4	***	0.0002	188.3	137.6 to 239.1	****	<0.0001
	(5E11).1107086_OD vs. (5E11).1107086_OG	106.8	61.16 to 152.4	****	<0.0001	211.9	166.3 to 257.5	****	<0.0001
	(5E11).1107086_OD vs. (5E11).1106077_OG	111.9	75.1 to 148.8	****	<0.0001	190.9	154 to 227.7	****	<0.0001
	(5E11).1107086_OD vs. (5E11).BB325g_OD	43.22	4.519 to 81.92	*	0.014	51.63	12.93 to 90.33	***	0.0008
	(5E11).1107086_OD vs. (5E11).BB325g_OG	114.3	63.52 to 165.1	****	<0.0001	211.8	161 to 262.6	****	<0.0001
	(5E11).1107086_OG vs. (5E11).1106077_OG	5.146	-45.96 to 56.25	ns	>0.9999	-21.02	-72.12 to 30.08	ns	0.9731
	(5E11).1107086_OG vs. (5E11).BB325g_OD	-63.56	-116 to -11.1	**	0.0043	-160.2	-212.7 to -107.8	****	<0.0001
	(5E11).1107086_OG vs. (5E11).BB325g_OG	7.551	-54.4 to 69.5	ns	>0.9999	-0.1058	-62.05 to 61.84	ns	>0.9999
	(5E11).1106077_OG vs. (5E11).BB325g_OD	-68.71	-113.7 to -23.68	****	<0.0001	-139.2	-184.3 to -94.2	****	<0.0001
	(5E11).1106077_OG vs. (5E11).BB325g_OG	2.405	-53.38 to 58.19	ns	>0.9999	20.91	-34.87 to 76.7	ns	0.987
	(5E11).BB325g_OD vs. (5E11).BB325g_OG	71.11	14.08 to 128.2	**	0.0027	160.1	103.1 to 217.2	****	<0.0001

Table S2. Results of Tukey's multiple comparison tests for the dose-response effect.

Only the results for the two highest light intensities are given. No significant differences were observed for the other three light intensities. One of the four retinas treated with a medium dose displayed no response to light, two had significantly weaker responses than 5 and 6 (of 8) of the retinas receiving a high dose and illuminated at the highest light intensity. Finally, only one single medium-dose retina ((5E10).BB161i_OG) had a significantly stronger response than three of the eight high-dose retinas, for the highest light intensity only, giving a positive mean difference (bold red). This retina also had a significantly weaker response than four of the high-dose retinas.

Supplementary References

1. Choi, V. W., Asokan, A., Haberman, R. A., McCown, T. J. & Samulski, R. J. Production of recombinant adeno-associated viral vectors and use for in vitro and in vivo administration. *Curr Protoc Neurosci* **Chapter 4**, Unit 4.17 (2006).
2. Aurnhammer, C. *et al.* Universal real-time PCR for the detection and quantification of adeno-associated virus serotype 2-derived inverted terminal repeat sequences. *Hum Gene Ther Methods* **23**, 18–28 (2012).
3. Szűcs, A. Applications of the spike density function in analysis of neuronal firing patterns. *Journal of Neuroscience Methods* **81**, 159–167 (1998).
4. Gauvain, G. & Murphy, G. J. Projection-Specific Characteristics of Retinal Input to the Brain. *J. Neurosci.* **35**, 6575–6583 (2015).
5. Quiroga, R. Q. & Panzeri, S. Extracting information from neuronal populations: information theory and decoding approaches. *Nat Rev Neurosci* **10**, 173–185 (2009).

6. Magri, C., Whittingstall, K., Singh, V., Logothetis, N. K. & Panzeri, S. A toolbox for the fast information analysis of multiple-site LFP, EEG and spike train recordings. *BMC Neuroscience* **10**, 81 (2009).
