# Supplementary material for: Optogenetic therapy: high spatiotemporal resolution and pattern discrimination compatible with vision restoration in non-human primates
Source: Commun Biol. 2021 Jan 27;4:125. doi: 10.1038/s42003-020-01594-w (PMC7840970; doi:10.1038/s42003-020-01594-w)
Supplement: Supplementary file 3 — Description of Additional Supplementary Files [file 42003_2020_1594_MOESM3_ESM.pdf]

Description of Additional Supplementary Files

Movie S1. RGC responses to spot stimulations

Spot stimuli of different sizes and presentation durations are displayed on the retina while recording the activity of individual RGCs on a grid of electrodes. Both the size and duration are written at the top of each panel. The black and white image shows the fluorescence present in the perifoveal area of the recorded retina explant, with the electrodes visible as black dots. Stimulations are located as orange dots, and neuronal responses are displayed as blue circles, the diameter of which are related to the spiking frequencies of individual cells (spike-sorted data).

Movie S2. RGC responses to moving bars

A 75 µm bar is presented in four different directions on the recorded retina. The video is slowed down five times but its real time is indicated at the top of the image. The black and white image shows the fluorescence present in the perifoveal area of the retina explants, with the electrodes visible as black dots. Stimulation is shown as an orange bar, and neuronal responses are displayed as blue circles, the diameter of which are related to the spiking frequencies of individual cells (spike-sorted data).

Movie S3. RGC responses to different moving shapes

Side-by-side comparison of the responses in the same retinal explant to two different shapes (circle and square). Shapes are displayed in orange and follow the same trajectory on the retina. The black and white image shows the fluorescence present in the perifoveal area, with electrodes visible as black dots. Neuronal responses are displayed as blue circles, the diameter of which are related to the spiking frequencies of individual cells (spike-sorted data).
