# Supplementary material for: Optogenetic therapy: high spatiotemporal resolution and pattern discrimination compatible with vision restoration in non-human primates
Source: Commun Biol. 2021 Jan 27;4:125. doi: 10.1038/s42003-020-01594-w (PMC7840970; doi:10.1038/s42003-020-01594-w)
Supplement: Supplementary file 7 — Reporting Summary [file 42003_2020_1594_MOESM7_ESM.pdf]

nature research

Corresponding author(s): Gregory Gauvain

Last updated by author(s): 23/11/20

Reporting Summary

Nature Research wishes to improve the reproducibility of the work that we publish. This form provides structure for consistency and transparency in reporting. For further information on Nature Research policies, see Authors & Referees and the Editorial Policy Checklist .

Statistics

For all statistical analyses, confirm that the following items are present in the figure legend, table legend, main text, or Methods section.

n/a	Confirmed	
☐	☒	The exact sample size (n) for each experimental group/condition, given as a discrete number and unit of measurement
☐	☒	A statement on whether measurements were taken from distinct samples or whether the same sample was measured repeatedly
☐	☒	The statistical test(s) used AND whether they are one- or two-sided<br>*Only common tests should be described solely by name; describe more complex techniques in the Methods section.*
☒	☐	A description of all covariates tested
☐	☒	A description of any assumptions or corrections, such as tests of normality and adjustment for multiple comparisons
☐	☒	A full description of the statistical parameters including central tendency (e.g. means) or other basic estimates (e.g. regression coefficient) AND variation (e.g. standard deviation) or associated estimates of uncertainty (e.g. confidence intervals)
☒	☐	For null hypothesis testing, the test statistic (e.g. F, t, r) with confidence intervals, effect sizes, degrees of freedom and P value noted<br>*Give P values as exact values whenever suitable.*
☒	☐	For Bayesian analysis, information on the choice of priors and Markov chain Monte Carlo settings
☒	☐	For hierarchical and complex designs, identification of the appropriate level for tests and full reporting of outcomes
☒	☐	Estimates of effect sizes (e.g. Cohen's d, Pearson's r), indicating how they were calculated

Our web collection on statistics for biologists contains articles on many of the points above.

Software and code

Policy information about availability of computer code

Data collection	All multielectrode array data was recording using MC rack software (Multichannel system), later extracted to matlab using MCS function suite. Confocal images were acquired using Fluoview software (Olympus).
Data analysis	Analysis and statistical testing were done using custom made matlab function or Graphpad Prism (version 7)

For manuscripts utilizing custom algorithms or software that are central to the research but not yet described in published literature, software must be made available to editors/reviewers. We strongly encourage code deposition in a community repository (e.g. GitHub). See the Nature Research guidelines for submitting code & software for further information.

Data

Policy information about availability of data

All manuscripts must include a data availability statement. This statement should provide the following information, where applicable:

- Accession codes, unique identifiers, or web links for publicly available datasets
- A list of figures that have associated raw data
- A description of any restrictions on data availability

The data that support the main findings of this study are openly available in figShare (https://figshare.com/projects/AAV2_7m8-ChrimsonR-tdTomato_for_vision_restoration/83675) as well as code repository Github (https://github.com/himstien/Optogenetic_Retinal_Data_Analysis).

Field-specific reporting

Please select the one below that is the best fit for your research. If you are not sure, read the appropriate sections before making your selection.

Life sciences study design

All studies must disclose on these points even when the disclosure is negative.

Sample size	We first performed a construct selection assay after 2 months of expression in vivo (8 animals, 4 eyes per construct tested). We then used our best construct to define optimal virus load in a long-term expression assay (6 months) with physiological and imaging methods (6 animals, 4 eyes per viral dose). We subsequently added 4 animals at high viral dose to extend our dataset. For a total of 18 animals.
Data exclusions	no data was excluded
Replication	all our findings were confirmed through replications
Randomization	Allocation of animals to experimental groups was random, serotype/dose injection in animals eye were not randomized in order to facilitate the injection procedure for the surgeon
Blinding	Sacrifice were performed in random order, tissues labeled with animal ID for experimenters until post-hoc analysis

Reporting for specific materials, systems and methods

We require information from authors about some types of materials, experimental systems and methods used in many studies. Here, indicate whether each material, system or method listed is relevant to your study. If you are not sure if a list item applies to your research, read the appropriate section before selecting a response.

Materials & experimental systems

n/a	Involved in the study	
☒	☐	Antibodies
☒	☐	Eukaryotic cell lines
☒	☐	Palaeontology
☐	☒	Animals and other organisms
☒	☐	Human research participants
☒	☐	Clinical data

Methods

n/a	Involved in the study	
☒	☐	ChIP-seq
☒	☐	Flow cytometry
☒	☐	MRI-based neuroimaging

Animals and other organisms

Policy information about studies involving animals; ARRIVE guidelines recommended for reporting animal research

Laboratory animals	18 macaca fascicularis, both sex, dage range 3 to 13 years (see table S1)
Wild animals	the study did not involve wild animals
Field-collected samples	the study did not involve samples collected from the field
Ethics oversight	All experiments were done in accordance with the National Institutes of Health Guide for Care and Use of Laboratory Animals. The protocol was approved by the Local Animal Ethics Committees and conducted in accordance with Directive 2010/63/EU of the European Parliament.

Note that full information on the approval of the study protocol must also be provided in the manuscript.
